# Thyroid-associated orbitopathy following COVID-19: a case report

**DOI:** 10.11604/pamj.2025.51.71.47807

**Published:** 2025-07-09

**Authors:** Sabrine Mekni, Hager Khiari, Manel Mekni, Jihene Sayadi, Sawsen Essayeh, Rihab Laamouri, Imen Rojbi, Ibtissem Ben Nacef

**Affiliations:** 1 Department of Endocrinology, Charles Nicolle Hospital, Tunis, Tunisia,; 2 Faculty of Medicine, University of Tunis El Manar, Tunis, Tunisia,; 3 Laboratory of Renal Pathology, LR00SP01, Charles Nicolle Hospital, Tunis, Tunisia,; 4 Department of Ophthalmology, Hedi Raies Hospital, Tunis, Tunisia

**Keywords:** Graves' ophthalmopathy, Graves' disease, SARS-CoV-2, iodine radioisotopes, case report

## Abstract

Studies have shown a link between COVID-19 vaccination and the development of Graves' disease (GD) and Graves' orbitopathy (GO). However, to the best of our knowledge, this is only the second reported case of clinically overt GO following COVID-19 infection. Herein, we report a 48-year-old female patient who was initially treated in 2017 with radioactive iodine therapy for a toxic thyroid adenoma. In 2021, the patient was diagnosed with COVID-19. Ten days later, she developed bilateral, active, moderate-to-severe GO, accompanied by elevated thyrotropin receptor antibody (TRAb) levels. She was treated with intravenous methylprednisolone followed by oral prednisone and later underwent orbital decompression surgery, followed by strabismus surgery due to worsening diplopia. No complications occurred postoperatively, and her symptoms improved significantly. This case contributes to the literature by suggesting a potential association between COVID-19 infection and the onset of autoimmune diseases, including GO.

## Introduction

Thyroid eye disease (TED) is an autoimmune inflammatory condition that affects the retroocular tissue. It is a debilitating and potentially sight-threatening condition. Most patients with TED (90%) have hyperthyroidism, and approximately 10% are euthyroid or hypothyroid [1]. Graves´ disease (GD) is the most common cause of TED (80%), known as Graves´ orbitopathy (GO). The disease is characterized by an active inflammatory phase followed by a stable and inactive chronic phase [1].

The etiopathogenesis of GD is multifactorial and involves a complex combination of genetic and non-genetic factors. This interplay results in the loss of immune tolerance to thyroid antigens and the initiation of a persistent autoimmune reaction. Infections, among other non-genetic factors, are believed to be crucial in triggering the disease process, particularly in individuals with a genetic susceptibility [2]. Since the COVID-19 pandemic began, millions of people have been affected, and the virus has been shown to cause a multitude of systemic disorders, including immune dysregulation such as autoimmune thyroiditis and GD [2,3]. Few cases of GD and GO after COVID-19 vaccination have been reported [4,5]. Some cases of new-onset GD after COVID-19 infection have been reported [3,6,7], but only one case presented with clinically overt GO [7]. To the best of our knowledge, this is the second reported case of clinically overt GO after COVID-19 infection that developed in a patient initially treated for hyperthyroidism due to TA. Our case contributes to the growing body of evidence in the literature suggesting a potential association between GO and COVID-19 infection.

## Patient and observation

**Patient information:** we report the case of a 48-year-old female patient with a history of hyperthyroidism and toxic thyroid adenoma (TA), who was treated with radioactive iodine (RAI) therapy in 2017. Four years later, she was referred to the endocrinology department for TED symptoms with inflammatory features. In February 2017, the patient presented with a nodule in the right thyroid. Thyroid ultrasound showed a 7-mm right nodule classified as European Thyroid Imaging-Reporting and Data System (EU-TIRADS) 3. Blood tests showed low thyroid-stimulating hormone (TSH) at <0.05 mIU/L (normal range 0.4-4) and an elevated free thyroxine (FT4) level of 1.82 ng/dL (normal range 0.7-1.5 ng/dL). Tests for thyroid peroxidase antibodies (TPOAb) and thyroid receptor antibodies (TRAb) were negative. Thyroid scintigraphy revealed a hyperfunctioning nodule ("hot"). The patient was initially treated with benzylthiouracil for three months, followed by RAI therapy in June 2017. She developed hypothyroidism in October 2017, was treated with 100 µg of levothyroxine daily, and had normal thyroid function at subsequent follow-up visits.

**Timeline of current episode:** the patient was diagnosed with COVID-19 in April 2021

**Clinical findings:** ten days later, she developed bilateral eye redness, eyelid fullness, and diplopia. The patient was referred to an ophthalmologist for a TED evaluation. He noted bilateral asymmetric exophthalmos, restrictive ocular dysmotility, and a clinical activity score of 4/7.

**Figure 1:**
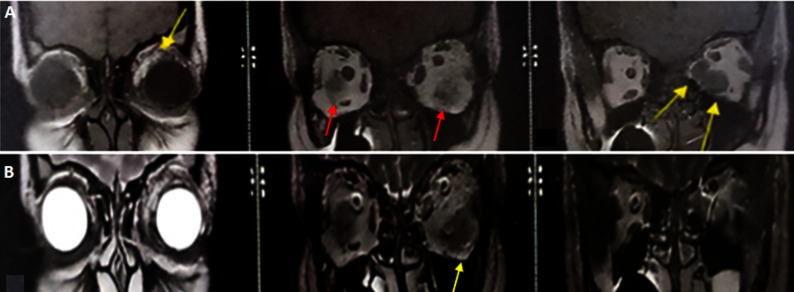
coronal T1-weighted (A) and T2-weighted (B) bilateral ocular proptosis grade I on the right side and grade II on the left side, enlargement and T2-weighted fat-saturated hyperintensity of the left inferior and medial rectus muscles and the left superior oblique muscle, measuring 10 mm, 6.5 mm, and 4 mm, respectively (yellow arrow), bilateral increased orbital fat volume more pronounced on the left (red arrow)

**Diagnostic assessment:** laboratory investigations revealed elevated TSH (8.17 mIU/l) and TRAb concentrations of 2.5 IU/L (normal range <2 IU/L). The results of the blood test are shown in Table 1. The coronal magnetic resonance imaging of the eye showed bilateral proptosis with enlargement of the left inferior and medial rectus muscles and left superior oblique muscle (Figure 1), indicating active, moderate-to-severe GO.

**Table 1:** hormonal and antibody assessments

Biological assessment	Result of the diagnosis of TA in 2017	Result after COVID-19 in 2021	Reference range
TSH (mIU/L)	<0.05	8.17	0.4-4
FT4 (ng/dL)	1.82	-	0.7-1.5
TPOAb (IU/L)	Negative	Negative	< 30
TRAb (IU/L)	Negative	2.5	< 2
Comparison of hormonal and antibody assessment results at the diagnosis of toxic thyroid adenoma (TA) in 2017 and following COVID-19 infection in 2021; TSH: thyroid-stimulating hormone; FT4: free thyroxine 4; TPOAb: thyroid peroxidase antibodies; TRAb: thyroid receptor antibodies			

**Diagnosis:** the diagnosis of overt GO following COVID-19 infection was made based on these findings.

**Therapeutic interventions:** the patient was treated with 125 µg of levothyroxine daily and intravenous methylprednisolone (1 g/day for three days), followed by oral prednisone (0.5 mg/kg/day) for three months. Significant improvement in inflammatory symptoms was observed, but diplopia worsened, which had an important negative impact on the patient´s quality of life. Once the GO became inactive, the patient underwent decompressive surgery of the orbits, followed by strabismus surgery consisting of recession of the retracted muscles to reduce restrictive dysmotility.

**Figure 2:**
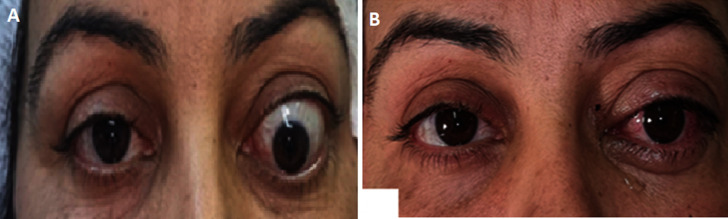
A) proptosis and strabismus of the left eye before surgery; B) proptosis and strabismus of the left eye after surgery

**Follow-up and outcome of interventions:** Figure 2 shows the evolution of strabismus after surgery. No complications occurred after the surgery.

**Patient perspective:** the patient was satisfied as her symptoms improved significantly.

**Informed consent:** written informed consent was obtained from the patient.

## Discussion

Our article presents a rare case of clinically overt GO after COVID-19 infection. Our case contributes to the literature by suggesting a potential association between GD and COVID-19 infection. The main limitation of our study is the report of a single case. Since the start of the COVID-19 pandemic, few cases of new-onset autoimmune thyroid disease have been reported following COVID-19 infection or vaccination [3]. It has been reported that 15% of patients with mild-to-moderate COVID-19 have thyroid dysfunction, and that SARS-CoV-2 can intensify pre-existing autoimmune thyroid disease [3]. Few cases of GD and GO have been reported after COVID-19 vaccination or infection [1,3,5,8,9]. To the best of our knowledge, only one case with clinically overt GO after COVID-19 infection has been observed, making this the second reported case [7].

Our patient was initially treated for hyperthyroidism secondary to TA. She developed hypothyroidism after RAI therapy and was treated with daily levothyroxine. Four years later, she developed TED ten days after the COVID-19 infection, suggesting that the COVID-19 infection plays a role. In fact, COVID-19 infection may act through several mechanisms. Molecular mimicry between viral and self-antigens is the first hypothesis, as various SARS-CoV-2 proteins share genetic similarity or homology with human proteins. COVID-19 activates B lymphocytes that produce antibodies against the virus. These antibodies might cross-react with thyroid antigens and periorbital tissues, causing GD and GO [9]. Furthermore, angiotensin-converting enzyme-2 (ACE2), the primary target of COVID-19, is highly expressed in the thyroid and is potentially implicated in the disease [2,7]. In addition, stimulation of the inflammasome by the release of type 1 interferon may lead to the production of TRAb and the development of GD [9]. Our patient initially tested negative for TRAb. After the infection, laboratory results showed elevated serum TRAb levels. This finding supports our hypothesis. The breakdown of central and peripheral tolerance, as well as psychological stress, may also contribute to the development of Hashimoto's thyroiditis (HT) and GD following COVID-19 infection [2,7].

Another interesting fact about our case was that the patient successively developed two different causes of hyperthyroidism: TA, which was specifically treated with RAI, and GD. GD is reported as an uncommon adverse effect after RAI therapy for TA and toxic multinodular goiter (MNG) [10]. The incidence of GD after RAI has been reported to be 5%, but it increases to 22% in patients who are positive for TPOAb before treatment. This was not the case in our patient, as TPOAb were negative in 2017. Several mechanisms have been proposed to explain the development of autoimmune hyperthyroidism after RAI. These include RAI-induced increases in circulating thyroid-stimulating immunoglobulins and radiation-induced disruption of the balance between T-helper cells and suppressor lymphocytes [10]. GD typically develops 3-6 months after RAI [10]. Our patient developed the condition four years later, which makes the causal link improbable.

Most reported cases of GO after the COVID-19 vaccine were moderate-to-severe and treated with immunosuppressants [1,3,5,8,9]. One case of vision-threatening GO and optic neuropathy after COVID-19 vaccination has been reported in the literature [9]. The patient was treated with immunosuppressants and underwent orbital decompression. Our patient had moderate-to-severe GO and was treated with intravenous corticosteroids. The inflammatory symptoms improved, but the diplopia worsened. As recommended, when the GO became an inactive form, decompressive orbital surgery was performed, followed by strabismus surgery.

## Conclusion

This paper outlines a rare case of severe GO that developed after COVID-19 infection, which was effectively managed with intravenous corticosteroids and subsequent surgical intervention. This case highlights a potential association between COVID-19 infection and the onset of GO, underscoring the importance for clinicians to consider this link when evaluating patients presenting with new autoimmune manifestations following COVID-19 infection.
